# Effect of Multiple Thermal Cycles on the Microstructure and Mechanical Properties of AISI 1045 Weldments

**DOI:** 10.1021/acsomega.2c05249

**Published:** 2022-11-09

**Authors:** Muhammad Atif Makhdoom, Furqan Ahmed, Iftikhar Ahmed Channa, Aqil Inam, Fahad Riaz, Sajid Hussain Siyal, Muhammad Ali Shar, Abdulaziz Alhazaa

**Affiliations:** † Institute of Metallurgy and Materials Engineering, University of the Punjab, Lahore 54590, Pakistan; ‡ Metallurgical & Materials Engineering Department, Faculty of Chemical, Metallurgical and Polymer Engineering, University of Engineering & Technology (UET), Lahore 54000, Pakistan; § Department of Metallurgical Engineering, NED University of Engineering and Technology, Karachi 75270, Pakistan; ∥ Department of Metallurgy and Materials Engineering, Dawood University of Engineering and Technology, Karachi 74800, Pakistan; ⊥ Department of Mechanical & Energy Systems Engineering, Faculty of Engineering and Informatics, University of Bradford, Bradford BD7 1DP, U.K.; # Department of Physics and Astronomy, College of Science, King Saud University, Riyadh 11451, Saudi Arabia

## Abstract

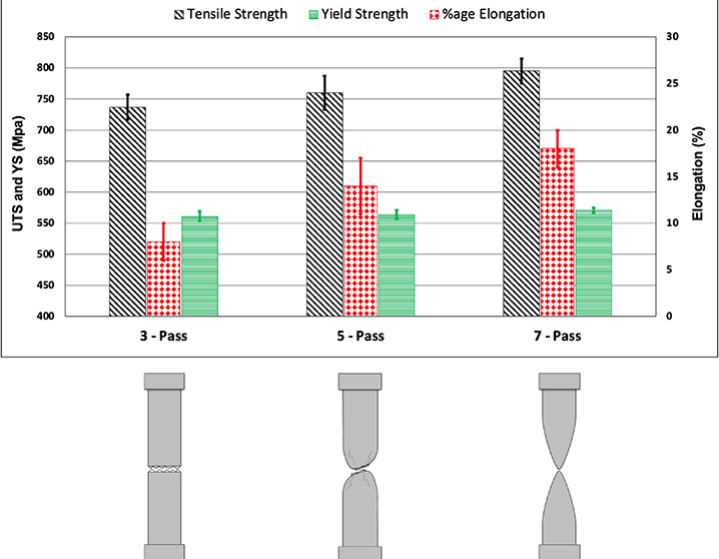

AISI 1045 medium carbon steel sheets having 10 mm thickness were subjected to the shielded metal arc welding process with three, five, and seven passes. The variations in the microstructure due to multiple thermal cycles in the heat-affected zone (HAZ), base metal (BM), and fusion zone (FZ) have been investigated and correlated with measured mechanical properties. Upon comparing fracture mechanics and mechanical properties with microstructural observations, it is elucidated that samples become ductile by increasing the number of thermal cycles which can be attributed to the transformations in the ferrite morphology in the HAZ. Based on mechanical, microstructural, and fracture analysis, it is concluded that post-weld heat treatment can be avoided if the number of passes during welding is increased.

## 1 Introduction

The induction of heat during multipass welding incorporates a heat-treatment effect in the microstructure which is analogues if the same sample is treated otherwise in a furnace under similar conditions. The high heating and cooling cycles,^1−3^ however, drastically change the microstructure in the fusion zone (FZ) and heat-affected zone (HAZ) during welding which is normally not observed after usual heat treatment processes in the same steel. High peak temperature during welding is the main reason for change which becomes more pronounced during multipass welding.^4−6^ In multi-pass welding, different zones are formed in the weldment based on microstructural changes which occur with temperature variation.

Medium carbon steels have found a wide range of engineering applications due to their better mechanical properties and good response toward heat treatment, for example, in shafts, pressure structures, bogie frames of railway vehicles, and so forth.^7−11^ In case of higher-carbon range medium carbon steels, the HAZ attains full hardness without any alloying addition which is more likely due to the formation of martensite. This endows weldments with low ductility and high susceptibility to hydrogen induction and cold cracking. The tendency of martensite formation in the HAZ during welding deteriorates the mechanical properties of medium carbon steel when compared to those of low carbon steel.^4^ However, martensite in the HAZ of the first weld pass is tempered by the heat ensuing from the deposition of subsequent passes.^12^ These microstructural changes arising during welding not only affect its electrochemical properties^13^ but also affect mechanical properties. These changes are difficult to simulate using computer-aided software which is otherwise very helpful in many engineering applications.^14^ This is due to lack of understanding of the phase transformations occurring within the material as a result of transient thermomechanical–chemical boundary conditions. This demands an in-depth knowledge of microstructural changes along with other parameters during welding.^15^ Therefore, the effect of the microstructure on the mechanical properties during welding has been the area of interest in contemporary research,^16−20^ but the literature on medium carbon steel is scanty in this particular direction and hence the present research. In this article, the effect of multi-pass welding on the microstructural variations in different welding zones of medium carbon steel has been investigated by eliminating the need for post-weld heat treatment (PWHT) which can save time and cost. The obtained results have been correlated with the mechanical properties and fracture mechanism.

## 2 Experimentation

**Figure 1:**
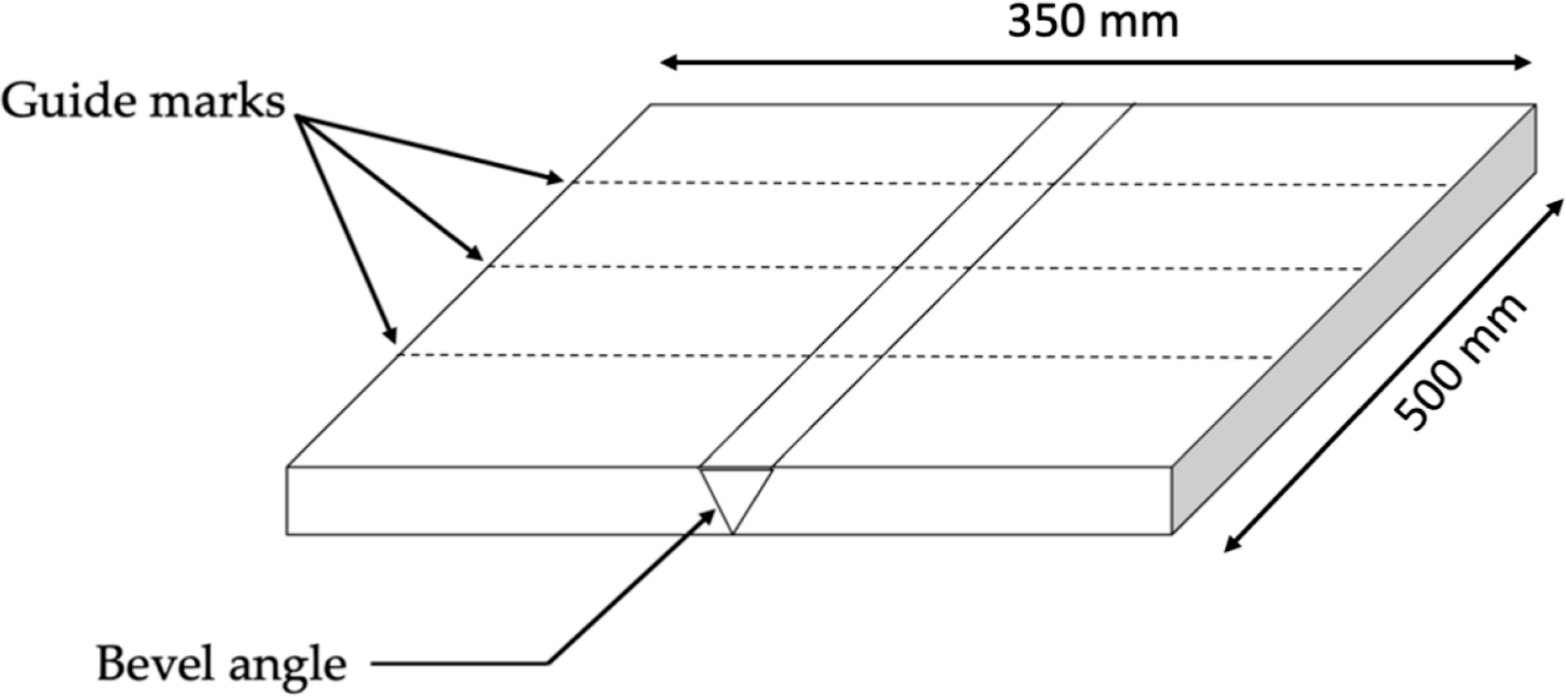
Schematic illustration of welding plates showing the guide marks and bevel angle.

Rolled plates of medium carbon steel AISI 1045 having 10 mm thickness were used as the base metal (BM). Bevel angles of 40, 60, and 80° were made for three, five, and seven passes, respectively. Butt weld joints with the designated filler metal (8018-B2) were made using the shielded metal arc welding (SMAW) process as per AWS codes and standards. To ensure heat input in each pass, marks were made along the length of the plate as shown schematically in Figure 1.

In this specially adapted technique, every marked segment was welded with a single electrode at constant speed which then lifted off as the next mark was reached. This technique helped avoid weld discontinuity which may otherwise be found in test specimens leading to ambiguous results. Chemical compositions and welding parameters are given in Tables 1–3.

**Table 1:** Nominal Chemical Composition (wt %, Balance Fe) of the Base Metal

C	Si	Mn	Cr	Ni	Mo	W	Nb	S	P
0.43	0.22	0.64	0.13	0.068	0.023	0.013	0.014	0.022	0.097

**Table 2:** Nominal Chemical Composition (wt %, Balance Fe) and Mechanical Properties of the Filler Metal (8018-B2)

C	Si	Mn	Cr	Mo	Sn	P	UTS, MPa
0.071	0.41	0.81	1.12	0.51	<0.005	<0.010	630

**Table 3:** Welding Data for Three, Five, and Seven Passes

number of pass	length of pass (mm)	inter-pass temperature (°C)	voltage (V)	current (Amp)	average travel speed between each mark (mm/min)	diameter of electrode (mm)	POL
Welding Data for Three Passes							
1	304	33	23	84	44	2.5	DCEP
2	304	46	25	95	65	2.5	DCEP
3	304	77	24	88	72	4.0	DCEP
Welding Data for Five Passes							
1	304	42	23	79	64	2.5	DCEP
2	304	68	25	103	64	2.5	DCEP
3	304	75	24	75	95	2.5	DCEP
4	304	59	24	83	74	2.5	DCEP
5	304	76	26	111	67	4.0	DCEP
Welding Data for Seven Passes							
1	304	33	22	84	75	2.5	DCEP
2	304	48	23	95	86	2.5	DCEP
3	304	49	23	95	83	2.5	DCEP
4	304	48	24	95	75	2.5	DCEP
5	304	48	24	95	86	2.5	DCEP
6	304	49	25	95	87	2.5	DCEP
7	304	79	25	88	89	4.0	DCEP

For optical microscopy, three samples were precisely sectioned from the BM, FZ, and HAZ of all three types of welded joints and subjected to the conventional metallographic sample preparation technique followed by etching in 3% Nital for 10 s.

To investigate the morphology of fracture, a fractographic study was carried out which has become a popular technique with the invention of scanning electron microscopy (SEM).^21^ Field-emission SEM (FESEM) is also reported in the literature^22^ for the said analysis. In this work, FEI Inspect S50 SEM was used to examine the fractured surface of tensile-tested samples and take micrographs at different magnifications. The river pattern, dimples, lamellar tearing, and so forth, can be revealed by the magnified view of the fracture surface^23^ which help conclude the breaking pattern. The fractography study of tensile- and impact-tested samples is already documented.^24^

Nine samples for each test, that is, tensile, bend, and V-notch Charpy impact, were marked as per ASTM-E8 and sectioned from all three welded plates to ensure the consistency in results. A wire-cut electric discharge machine (EDM) was used to avoid any further changes in the microstructure which may otherwise occur due to heat generation in traditional cutting processes.

Bend (as per ASTM A36) and tensile tests were performed using Shimadzu UTM, model: UMH-200A T.V. having 200 ton capacity, while the impact test was performed on a Laryee Technology machine, model: CMT 2230.

## 3 Results and Discussion

**Figure 2:**
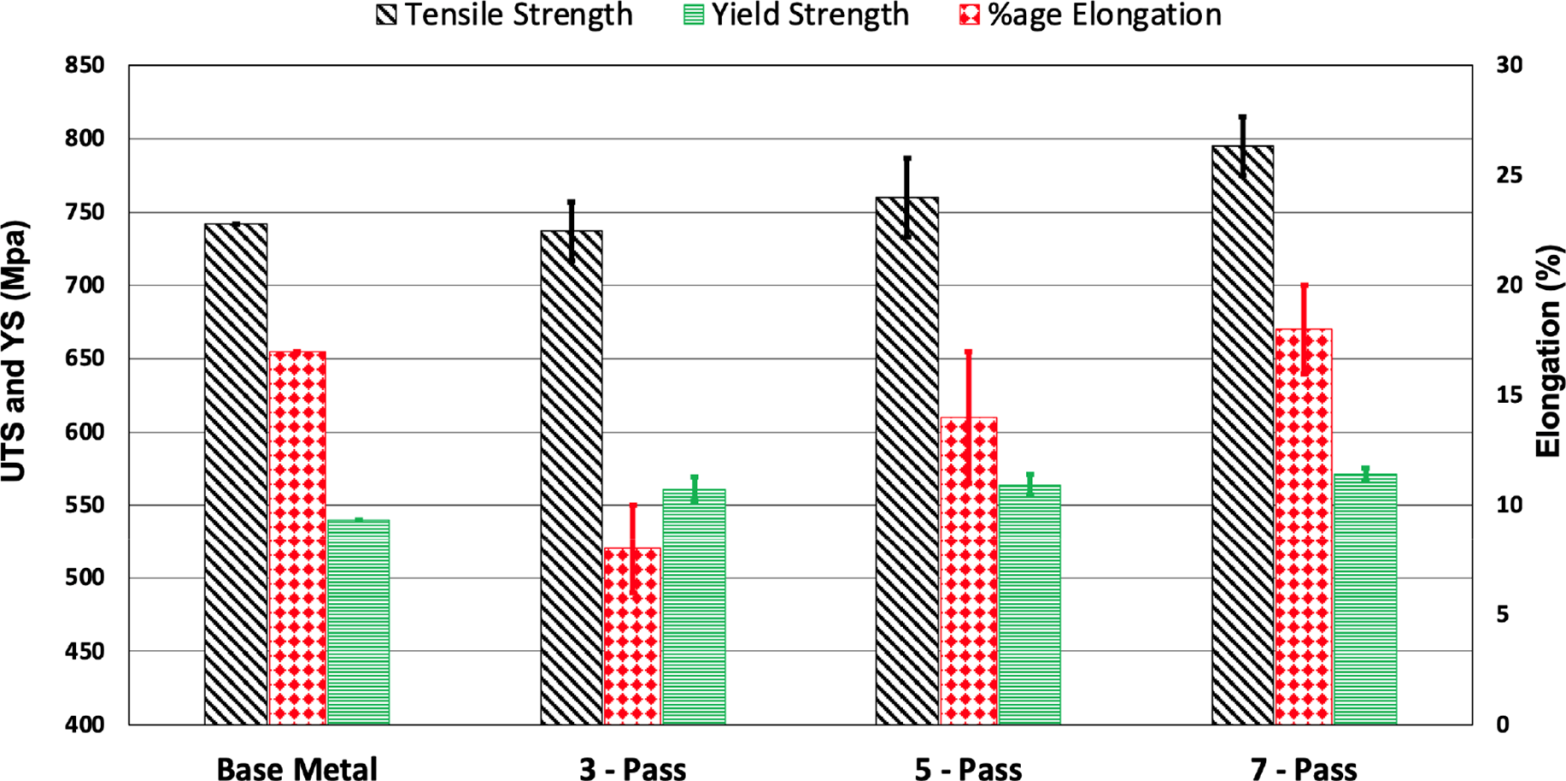
Average comparative values of tensile strength, yield strength, and percentage elongation for the BM and three, five, and seven passes.

**Figure 3:**
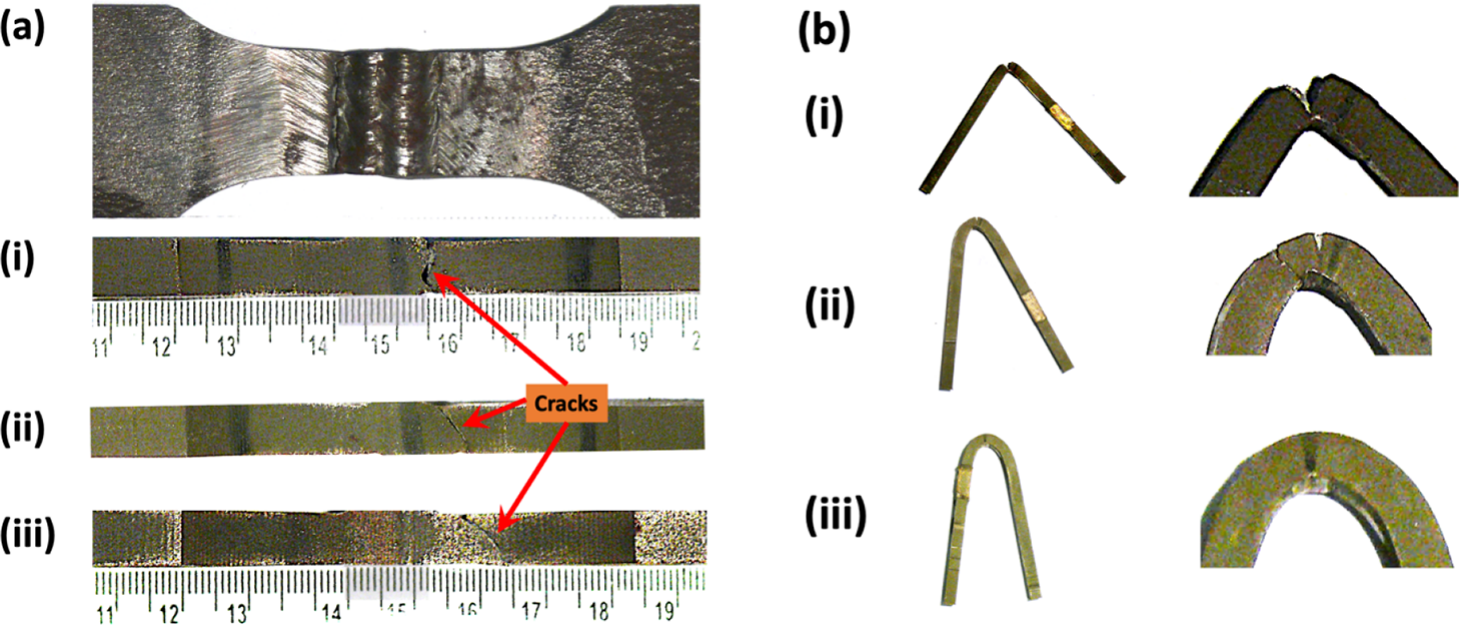
Side views of (a) tensile-tested samples showing fracture surface morphologies and (b) bend-tested samples with higher magnifications (right) showing crack formation after (i) three, (ii) five, and (iii) seven passes.

Figure 2 shows results of tensile strengths, yield strengths, and percentage elongation for all three passes. Results show that both ultimate tensile strength (UTS) and yield strength (YS) possess an increasing trend with the number of passes. The UTS of un-welded AISI 1045 steel is 742 Mpa, whereas for the samples subjected to three passes, the UTS was slightly reduced by 0.67%. However, for the samples subjected to five and seven passes, the UTS was increased by 2.42 and 7.14%, respectively. The percentage of elongation was also increased with the number of passes. All the samples were fractured from the HAZ. However, the side view of the fractured samples depicts that by increasing the number of passes, the fracture plane changes its direction from perpendicular to an angle of approximately 45°. This is a typical behavior of brittle to ductile transition—see Figure 3a(i–iii). For reference, the same sample is placed at the top in the horizontal position to differentiate FZ and HAZ areas. Here, it is pertinent to note that the said picture of the reference sample is of before removal of the weld reinforcement (capping pass) prior to testing.

**Figure 4:**
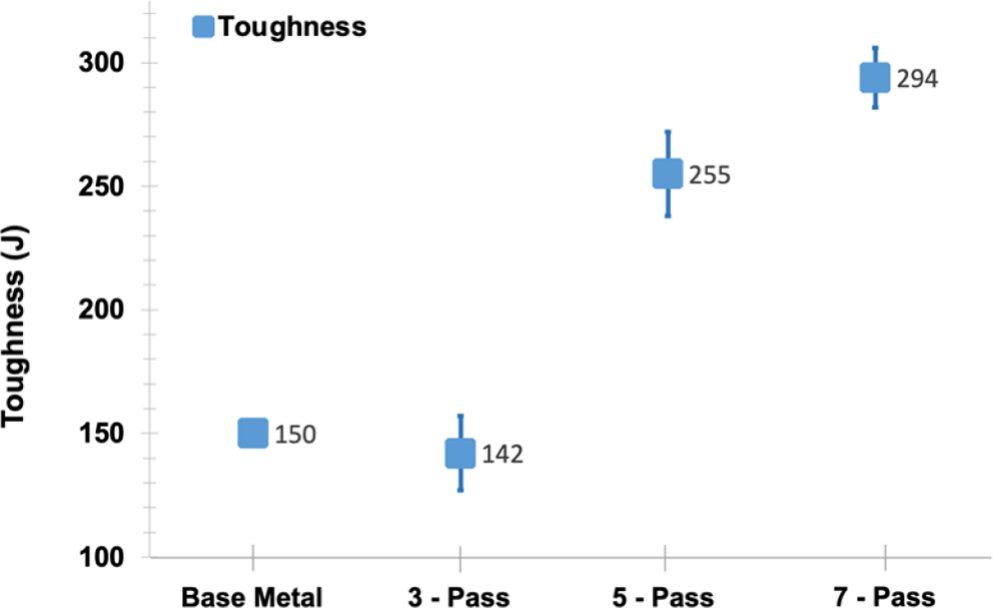
Average values of toughness of the BM and samples after three, five, and seven passes.

The bend test results under three-point load conditions show that samples with three and five passes failed to qualify the test—see Figure 3b(i,ii). High-magnification images show that fracture occurred at the HAZ. However, the samples subjected to seven passes qualified the test, and there was no fracture evident in the samples—see Figure 3b(iii). Figure 4 shows the results of Charpy V-notch absorbed energy versus number of passes. The graph exhibits an increase in the absorbed energy with the number of passes. The toughness of the bare AISI 1045 steel is 150 joules, but the toughness of the samples subjected to three passes was slightly decreased by 5.33%. However, toughness of the samples subjected to five and seven passes was tremendously increased by 70 and 96.67%, respectively. It has been reported that toughness of the FZ is lower than that of the BM and HAZ,^25,26^ but it is opposite in our case. This can be attributed to the number of thermal cycles that increase linearly with the number of passes which eventually modifies the previously formed microstructure. This phenomenon was investigated by analyzing the microstructures as explained in detail in the following sections.

**Figure 5:**
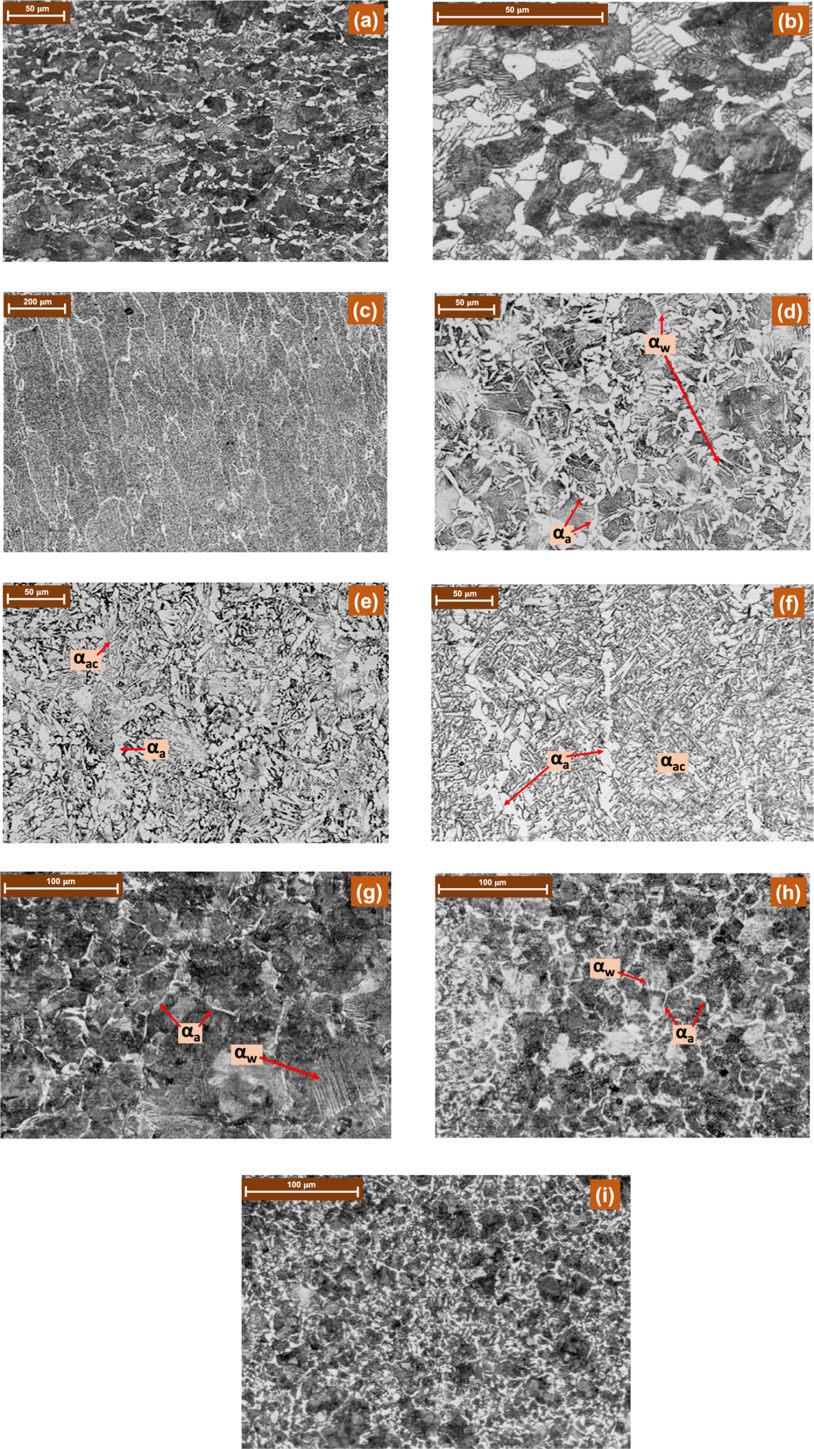
Microstructures of (a) BM at lower magnification, (b) BM at higher magnification, (c) FZ at lower magnification, (d) FZ at higher magnification of three passes, (e) FZ at higher magnification of five passes, (f) FZ at higher magnification of seven passes, (g) HAZ of three passes, (h) HAZ of five passes, and (i) HAZ of seven passes.

The microstructure of the BM—see Figure 5a—shows a combination of the pearlitic (black) and allotriomorphic ferrite (αa) (white) microstructure with a ratio of approximately 60:40 when observed at higher magnification—see Figure 5b.

Microstructures of the FZ of all the passes have a typical columnar morphology—see Figure 5c—but the grain size is enlarged as the number of passes increases from three to seven. At higher magnifications—see Figure 5d–f—the FZ shows the presence of ferrite at grain boundaries as allotriomorphic ferrite, Widmanstätten ferrite (αw), and acicular ferrite (αac). Widmanstätten ferrite is more pronounced in the FZ of three passes and with a less tendency in the FZ of five passes. In the FZ of seven passes, Widmanstätten ferrite is completely eliminated with the formation of acicular ferrite—see Figure 5f—which is comparatively lesser in the FZ of five passes and completely absent in the FZ of three passes.

Analysis of HAZ microstructures—see Figure 5g–i—clearly reveals a linear increase in the number of fine grains as the number of passes increases from three to seven. Coarse-grained pearlite in the three-pass HAZ—see Figure 5g—is encapsulated by allotriomorphic ferrite as grain boundary ferrite along with Widmanstätten ferrite which is typically nucleated at alpha–gamma grain boundaries and extended into untransformed austenite grain interiors.^27^ The amount of Widmanstätten ferrite is reduced or becomes almost negligible when the number of passes is increased from three to five with the refinement of coarse-grained pearlite—see Figure 5h. This can be attributed to multiple thermal cycles which are higher in case of five passes. This fact is further amplified as the number of passes is increased from five to seven, producing fine-grained pearlite with a high volume fraction of equiaxed polygonal ferrite—see Figure 5i.

The microstructure of the BM—see Figure 5a,b—is comparatively composed of a less amount of ferrite when compared with that of the HAZ and FZ. During welding, the temperature of the BM at a distance from the FZ remains below 200 °C, and hence, no microstructural variations occur.^4^

The number of thermal cycles with subsequent passes increases the austenite grain size which, in turn, reduces the grain boundary areas which are actually the preferential sites for the nucleation of proeutectoid ferrite, and therefore, coarsening of ferrite grain size occurs in the FZ with the number of passes increasing from three to seven. Moreover, less nucleation sites were available for pearlite nucleation due to limited availability of the ferrite–austenite interface which resulted in reduction of the pearlite volume fraction with the number of passes. The percent dilution of BM composition with the 8018-B2 filler metal also resulted in the formation of a large volume fraction of ferrite with different morphologies in the FZ. The nucleation of acicular ferrite occurs mostly at non-metallic inclusions.^28,29^ The addition of “Mo” from the filler metal also tended to preclude pro-eutectoid ferrite and favored acicular ferrite. The significant density of these nucleation sites in the FZ resulted in the formation of such a microstructure instead of banite^30,31^ and hence promoted strength and toughness of the material.^32^ However, the presence of allotriomorphic ferrite as grain boundary ferrite is unfavorable for the toughness,^33^ and a brittle fracture mode is more likely which is proportional to the amount of Widmanstätten ferrite that forms within the microstructure of the weldment along with allotriomorphic ferrite.^32,34^ Therefore, upon considering the combined effect of pearlite grain size, relative volume fractions of ferrite–pearlite phases, and the presence of a large amount of Widmanstätten ferrite along with allotriomorphic ferrite in the three-pass HAZ—see Figure 5g—and FZ—see Figure 5d, a brittle fracture is registered.

As the number of passes was increased, the transition from the brittle to the ductile fracture mode was observed with improved mechanical properties. The following reasons can be attributed to these observations: (i) the transformation of Widmanstätten ferrite to other forms of ferrite with subsequent thermal cycles in five and seven passes, see Figure 5g,h, and (ii) reduction in the grain size of the pearlite with the formation of a large amount of polygonal ferrite.

Mechanical tests showed that the strength of the weldments improved with the number of passes, but the region of the HAZ proved to be the weakest part as the weldments failed from here. The area of the HAZ is reheated during multipass welding which results in the development of different phases. It is reported that formation of banite in the HAZ in low carbon steel results in a brittle fracture,^35^ but here, in case of medium carbon steel, such a structure is not evident in subsequent passes.

**Figure 6:**
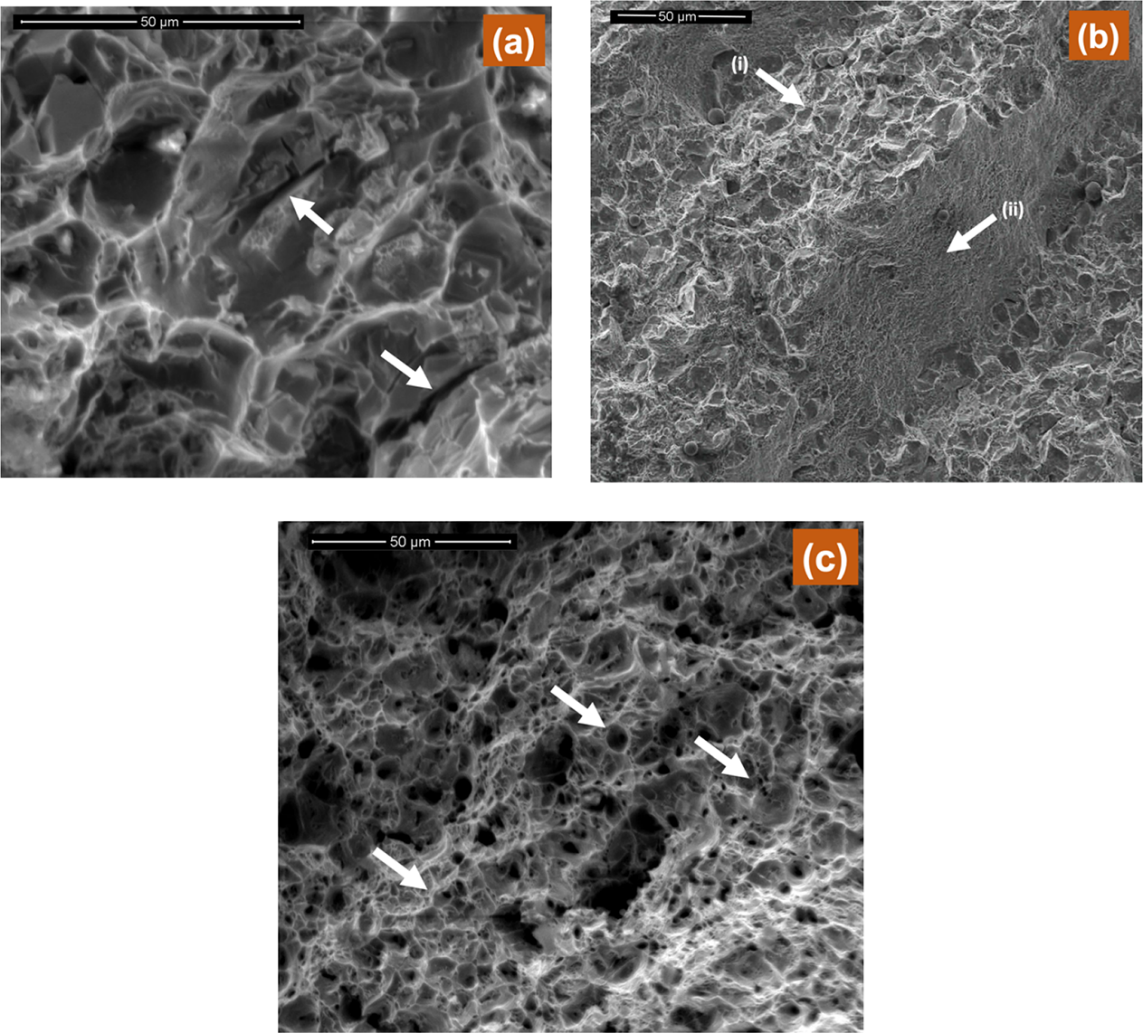
Fractographs of (a) three passes, (b) five passes, and (c) seven passes.

Figure 6 shows the fractographs taken with SEM from fractured surfaces after tensile tests. Figure 6a clearly shows that a typical cleavage-type transgranular brittle fracture which is evident from broken grains or cleavage facets and cracks—see white arrows in Figure 6a—is present on the fracture surface. The presence of Widmanstätten ferrite in the three-pass weldment provides suitable sites to initiate the trans-granular cracks, which are further augmented by the presence of large grains of pearlite. This has reduced overall toughness of the sample. Ductile fracture was found in the samples subjected to seven passes which is evident from the typical fibrous surface consisting of a considerable number of dimples/micro-voids—see white arrows in Figure 6c. Smaller size of dimples is associated with grain refinement. Elongated and coalesced dimples are the indicator of high plasticity and elongation as evident from the results of mechanical tests. Figure 6b, however, shows mixed fracture where cleavage fracture—see white arrows (ii) in Figure 6b—and shear dimples—see white arrows (i) in Figure 6b—both can be seen.

## 4 Conclusions

The SMAW process was carried out on medium carbon steel with three, five, and seven passes followed by mechanical characterization without PWHT. Following inferences are made from the current investigations:

- Reduction in grain size with the increasing number of passes is evident in the HAZ, resulting in the improvement in UTS and toughness of the weldment by approximately 7.14 and 96.67%, respectively.
- Multipass welding option can be adopted without PWHT in medium carbon steels wherever high toughness is required without compromising UTS.
- Formation of a high-volume fraction of polygonal ferrite and the absence of Widmanstätten ferrite in the seven-pass weldment demonstrated ductile fracture which was otherwise brittle in the three-pass weldment, where a comparatively higher volume fraction of Widmanstätten ferrite and large grain size were present.
- Fractographic study of the samples showed a systematic variation in the microstructure resulting in transition from transgranular brittle to ductile fracture with a mixed fracture in the five-pass weldment. These observations are also evident from tensile, bend, and toughness results and hence justify the fracture analysis.
